# Commentary: Ozone therapy as a novel complementary therapeutic approach in refractory idiopathic granulomatous mastitis

**DOI:** 10.3389/fmed.2023.1272059

**Published:** 2023-12-11

**Authors:** Salvatore Chirumbolo, Marianno Franzini, Sergio Pandolfi, Umberto Tirelli, Luigi Valdenassi

**Affiliations:** ^1^Department of Engineering for Innovation Medicine, University of Verona, Verona, Italy; ^2^Italian Scientific Society of Oxygen Ozone Therapy (SIOOT) and High Master School in Oxygen Ozone Therapy, University of Pavia, Pavia, Italy; ^3^Tirelli Medical Group, Pordenone, Italy

**Keywords:** mastitis, ozone, ozone therapy, SIOOT, protocols

## Introduction

A recent contribution by Cabiouglu et al. to this journal reported the successful ability of ozone to contribute to the treatment of refractory idiopathic granulomatous mastitis (IGM) (1). Idiopathic granulomatous mastitis (IGM) is a rare breast disorder characterized by the development of non-caseating granulomas within the breast tissue, where “idiopathic” means that the exact cause of the condition is unknown. It predominantly affects women of childbearing age, typically occurring in their 20s−40s, although it can be met at any age. The exact cause of idiopathic granulomatous mastitis remains poorly understood, but it is believed to involve an abnormal immune response within the breast tissue (2–4).

The manuscript is particularly interesting because it shows the ability of ozone therapy, as an adjunct treatment to corticosteroid therapy, to rescue patients from exacerbations and recover patients' health with success.

From a methodological point of view, we have some comments to be forwarded and possibly debated. In their report, the authors did not specify their “odd” concentration measure, μg/Nml, for which the reader cannot be sure if 15 or 30 μg/Nml corresponds to 15 or 30 μg/ml ozone (1): what the authors intended for “normalized”? If such, we wondered why the authors considered 30 μg/ml as an “immunosuppressive” dose; if they did not report any evidence regarding the immunosuppression led by 30 μg/ml, probably they intended “anti-inflammatory”, as well (1).

Actually, we wondered if this unusual way to indicate a concentration measure should be merely intended as 30 μg/ml or not. The authors should have explained their indications better.

Moreover, considering that the authors used effectively 30 μg/ml ozone via major autohemotherapy, it would be useful to know if this production was held at room temperature (between 20°C and 25°C), because the solubility of ozone in blood (37°C) is completely different with respect to oxygen and depends on the temperature, inasmuch as 30 μg/ml v/v (O_3_/O_2_) corresponds, according to Henry's law, to about 7.08 μg/ml into the plasma water, which is yet close to the anti-inflammatory range exhibited by the major O_3_-derived ozonide, 4-hydroxynonenal (4-HNE) (5). In addition, the authors also used minor autohemotherapy (1).

We observed, with a positive consideration, that 30 μg/ml may induce a plasma concentration of 4-HNE able to induce an anti-inflammatory response (5). However, our SIOOT/Multiossigen protocols prefer to reach a dose of approximately 40–50 μg/ml, in order to ensure the achievement of about 10 μg/ml. 4-HNE has been reported to exert effective anti-inflammatory activity toward the NLRP3 inflammasome (5).

Although Bocci et al. recommended an ozone range of 20–80 μg/ml, 30 μg/ml is within this range, using 40–50 μg/ml (usually 45 μg/ml) as closest to the range median and more reliable to reach a final dose of 3.0 μM 4-HNE without falling into bias in the ozone preparation (6). Moreover, the dose of 40 μg/ml O_3_ was considered by authors elsewhere for mastitis (7).

The evidence reported by the authors in using ozone as an adjunct therapy to cortisone is particularly encouraging anyway. Approximately 37.5% (*p* = 0.002) showed a complete response rate, and only 21% (*p* = 0.001) and 29.9% (*p* = 0.002) had recurrence at 1 year and 2 years following ozone therapy, respectively (1). In these successful cases, the use of corticosteroids was progressively reduced (1).

However, we discovered another error in this statistical calculation. In fact, we wondered whether these “complete response rates” were quantitative data in order to support the Mann-Whitney test; probably, they were not. Yet, considering the “absence” and “presence” of a complete response as a score (evaluated in SPSS v 24.0), a Kruskal-Wallis evaluation of the complete response rates should give *p* = 0.02298 (H = 5.1702). As a result, we can consider the evidence presented by the authors in IGM to be correct.

The bewildering action of ozone in IGM, despite the fact that this compound is notoriously toxic, should promote further insightful investigations to elucidate its biomolecular action. In this context, the authors limited their discussion to general aspects on the immunoregulatory role of ozone in IGM.

We would like to add some considerations of ours.

A role in IGM has been recently attributed to the cytokines IL-8 and IL-17 (8). Fundamentally, recent data would suggest that ozone reduces IL-17-producing cells, i.e., Th17 lymphocytes, and the expression levels of IL-17 and RORγt (9), reducing the impact of IGM if in IGM IL-17 is overexpressed (10), despite some controversial results (11). The anti-oxidant role attributed to ozone, inasmuch as it activates the Nrf2/Keap1/ARE pathway, can elucidate the ability of low doses of ozone to trigger an anti-inflammatory response (12).

Medicine should emphasize the crucial role of hormetic mechanisms, led by xenobiotics and involving mitochondria and the inflammasome NLRP3 (5, 13), in the step way climbing toward a successful therapy for major concerning pathologies.

Hormesis is a biological phenomenon where exposure to low doses of a stressor or harmful agent can result in beneficial effects such as improved resilience, increased lifespan, or enhanced physiological function. It is essentially a biphasic dose-response relationship, where the response to a stressor is opposite at low doses compared to higher doses. In other words, a little bit of a harmful agent might actually be good for an organism, while larger amounts can be detrimental. This mechanism, when regarding ozone, can promote the activity of reactive oxygen species (ROS) as signaling molecules, able to modulate the Nrf2-mediated response to inflammation and display an anti-inflammatory response (12, 13).

The beneficial activity of ozone in IGM may lie in its ability to induce a hormetic mechanism of immune modulation, either via Nrf2 or via 4-HNE and NLRP3 (5).

## Author contributions

SC: Conceptualization, Methodology, Supervision, Validation, Visualization, Writing – original draft, Writing – review & editing. MF: Supervision, Validation, Visualization, Conceptualization, Investigation, Writing – review & editing. SP: Data curation, Supervision, Validation, Visualization, Investigation, Writing – review & editing. UT: Data curation, Formal analysis, Investigation, Software, Supervision, Validation, Visualization, Writing – review & editing. LV: Formal analysis, Project administration, Resources, Supervision, Validation, Visualization, Writing – review & editing.
